# Work of breathing at different tidal volume targets in newborn infants with congenital diaphragmatic hernia

**DOI:** 10.1007/s00431-022-04413-y

**Published:** 2022-03-18

**Authors:** Rebecca Lee, Katie A. Hunt, Emma E. Williams, Theodore Dassios, Anne Greenough

**Affiliations:** 1 Department of Women and Children’s Health, School of Life Course Sciences, Faculty of Life Sciences and Medicine, King’s College London, London, UK; 2 Neonatal Intensive Care Centre, King’s College Hospital NHS Foundation Trust, 4th Floor Golden Jubilee Wing, Denmark Hill, SE5 9RS London, UK; 3 The Asthma UK Centre for Allergic Mechanisms in Asthma, London, UK; 4 NIHR Biomedical Research Centre based at Guy’s and St Thomas NHS Foundation Trust and King’s College London, London, UK

**Keywords:** Congenital diaphragmatic hernia, Volume targeted ventilation, Work of breathing, Pressure time product

## Abstract

Congenital diaphragmatic hernia (CDH) results in varying degrees of pulmonary hypoplasia. Volume targeted ventilation (VTV) is a lung protective strategy but the optimal target tidal volume in CDH infants has not previously been studied. The aim of this study was to test the hypothesis that low targeted volumes would be better in CDH infants as determined by measuring the work of breathing (WOB) in CDH infants, at three different targeted tidal volumes. A randomised cross-over study was undertaken. Infants were eligible for inclusion in the study after surgical repair of their diaphragmatic defect. Targeted tidal volumes of 4, 5, and 6 ml/kg were each delivered in random order for 20-min periods with 20-min periods of baseline ventilation between. WOB was assessed and measured by using the pressure–time product of the diaphragm (PTPdi). Nine infants with a median gestational age at birth of 38 + 4 (range 36 + 4–40 + 6) weeks and median birth weight 3202 (range 2855–3800) g were studied. The PTPdi was higher at 4 ml/kg than at both 5, *p* = 0.008, and 6 ml/kg, *p* = 0.012.

*Conclusion*: VTV of 4 ml/kg demonstrated an increased PTPdi compared to other VTV levels studied and should be avoided in post-surgical CDH infants.

**Table:** 

**What is Known:** *• Lung injury secondary to mechanical ventilation increases the mortality and morbidity of infants with CDH.* *• Volume targeted ventilation (VTV) reduces ‘volutrauma’ and ventilator-induced lung injury in other neonatal intensive care populations.*	
**What is New:** *• A randomised cross-over trial was carried out investigating the response to different VTV levels in infants with CDH.* *• Despite pulmonary hypoplasia being a common finding in CDH, a VTV of 5ml/kg significantly reduced the work of breathing in infants with CDH compared to a lower VTV level.*

**What is Known:**

*• Lung injury secondary to mechanical ventilation increases the mortality and morbidity of infants with CDH.*

*• Volume targeted ventilation (VTV) reduces ‘volutrauma’ and ventilator-induced lung injury in other neonatal intensive care populations.*

**What is New:**

*• A randomised cross-over trial was carried out investigating the response to different VTV levels in infants with CDH.*

*• Despite pulmonary hypoplasia being a common finding in CDH, a VTV of 5ml/kg significantly reduced the work of breathing in infants with CDH compared to a lower VTV level.*

## Introduction

Congenital diaphragmatic hernia (CDH) has an estimated incidence of 2.3 in 10,000 live births [1]. It is characterised by a defect in the diaphragm through which abdominal contents herniate and create a mass effect within the thorax which restricts normal development of the lungs, resulting in a reduction in the number of alveoli in addition to impeding the development of normal pulmonary vascular structures [2]. There have been many fetal and neonatal advances in care of infants with CDH over recent decades [3], which has resulted in improved survival [4]; however, there remains a high burden of morbidity [5] associated with the condition.

Historically, infants with CDH may have received aggressive ventilation strategies to provide lifesaving support resulting in ventilator-induced lung injuries due to high ventilator pressures [6], or complications associated with ventilatory-induced hypocarbia [7, 8]. Advances in care and prioritisation of the pre-operative stabilisation of infants with CDH have resulted in current management focused on the delayed surgical repair and ‘gentle ventilation’ to reduce pulmonary complications and improve survival and long-term morbidity [9]. The European Consortium consensus statement recommends that conventional ventilation should be used as a first line in infants with CDH [10]. In other neonatal intensive care populations, ventilator-induced lung injury has been proven to be minimised by utilising volume-targeted ventilation (VTV) to reduce ‘volutrauma’ of the lungs [11]. Furthermore, in infants born at or near term, higher VTV levels (5 and 6 ml/kg) compared to lower VTV levels (4 ml/kg) have been shown to reduce the work of breathing [12]. Interestingly, a recent systematic review and care pathway description reported that none of the protocols they looked at included VTV, perhaps due to a lack of evidence as how to apply it [13].

Pulmonary hypoplasia is a cardinal finding in CDH; we therefore postulated that the optimal targeted tidal volumes in infants with CDH would be smaller than in those used in term infants with other conditions [12]. Our aim, therefore, was to measure post-operatively the work of breathing at three different tidal volumes that are used in clinical practice (4, 5, and 6 ml/kg).

## Materials and methods

A randomised cross-over study of infants born with CDH at King’s College Hospital NHS Foundation Trust, London, UK, was undertaken. Infants were eligible for inclusion if they had undergone operative repair of CDH and were enrolled following written informed parental consent.

### Statement of ethics

The study was approved by the London – Camden and King’s Cross Research Ethics Committee and the Health Research Authority (16/LO/0887), and the UK Health Research Authority (HRA) (IRAS project ID: 201801).

Infants were studied when they were ventilated and not receiving muscle relaxants. Set target tidal volumes of 4, 5, and 6 ml/kg were each delivered in a random order, selected by a random number generator, for 20-min periods, with an additional 20 min of baseline pressure-limited ventilation in between each period of VTV. The positive end-expiratory pressure level and the back-up respiratory rate set on the ventilator were kept the same throughout the study. The fraction of inspired oxygen concentration (FiO_2_) was adjusted to maintain oxygen saturations between 92 and 96%.

The primary outcome of the work of breathing (WOB) was assessed using the pressure–time product of the diaphragm (PTPdi) method at the end of each set VTV period and period of baseline pressure limited ventilation. PTPdi is a correlative measure of oxygen consumption of the respiratory muscles [14] and an indicator of respiratory muscle energy expenditure [15].

Gastric and oesophageal pressures were measured using a dual pressure transducer tipped catheter (Gaeltec, Dunvegan, Scotland). Flow was assessed using a pneumotachograph (Mercury F10L; GM Instruments, Kilwinning, Scotland), which was inserted between the ventilator circuit and the endotracheal tube and connected to a differential pressure transducer (± 2 cm H_2_O; MP45; Validyne, Northridge, CA, USA). The pneumotachograph had a side port by which airway pressure was measured; this was connected to a pressure transducer (± 100 cm H_2_O; MP45; Validyne, Northridge, CA, USA). Air flow, airway pressure, and gastric and oesophageal pressure signals were recorded simultaneously on a computer running specially written software (Labview V.5.0, National Instruments, Austin, TX, USA) with 100 Hz analogue-to-digital sampling (16 bit DAQ card, DAQ 6036E, National Instruments, Austin, TX, USA). Tidal volume was calculated by digital integration of the flow signal by the software. Infants were ventilated using the SLE 5000 or 6000 ventilator (SLE, Croydon, UK). The primary ventilation mode was synchronised intermittent mandatory ventilation or patient-triggered ventilation and the PTPdi was measured for the supported breaths with a back-up rate of 30/min.

Transdiaphragmatic pressure was obtained by subtraction of the oesophageal pressure from the gastric pressure; this was then integrated with time for the inspiratory portion of each breath to give the PTPdi. For each breath, the beginning and end of inspiration was determined from the flow signal, in order to delineate the inspiratory work of breathing. The mean PTPdi of the first 20 artefact-free breaths in the last 5 min of each 20 min epoch of ventilation was calculated, as has previously been described [16].

#### Sample size

In the absence of previously published data on the PTPdi in infants with CDH, we based our sample size calculation on a clinically significant difference in the PTPdi of 68 cmH_2_O s/min in infants who were ventilated with synchronised intermittent positive pressure ventilation with targeted volumes of 4 and 6 ml/kg [17]. The reported standard deviation of the PTPdi was 59 cmH_2_O s/min. In order to detect a difference of 68 cmH_2_O s/min with 90% power and at a level of significance of 0.05 the intended sample size consisted of 16 infants. An interim analysis was planned to take place half way through, as our studies with different targeted volumes demonstrated that the PTPdi was better in all infants at 5 and 6 ml/kg compared to 4 ml/kg [17]. In order to preserve the type I error at 5%, the interim analysis was conducted at 0.01 with the final analysis conducted using 0.04. This gave an overall type 1 error rate (significance level) of 5% ((1–0.01) × (1–0.04) = 0.95 = 1–0.05). If the interim analysis showed *p* < 0.01, then the trial was to stop and the final analyses conducted using the patients treated to that point.

#### Statistical analysis

The data were tested for normality using the Shapiro–Wilk and D’Agostino skewness tests and found to be not normally distributed and are thus presented as median (range). A Friedman test was used to assess for differences between the PTPdi at different levels of volume targeting. Post hoc analysis was undertaken with Wilcoxon signed-rank tests with Bonferroni correction for multiple comparisons used. The statistical analysis was performed using SPSS software, version 26.0 (IBM, Armonk, NY, USA).

## Results

The interim analysis was performed at a sample size of nine infants as one more infant was recruited before the interim analysis could be performed. At the interim analysis, the comparison of the PTPdi at the different levels of volume targeting was statistically significant using the modified cut-off for significance described above. The PTPdi was lower at 5 and 6 ml/kg compared to 4 ml/kg for all infants; hence, the investigators agreed that the trial be stopped at that point and the data analysed.

Nine infants with a median gestational age at birth of 38 + 4 (range 36 + 4–40 + 6) weeks and median birth weight 3202 (range 2855–3800) g were studied. They underwent operative repair at a median of 4 days after birth (range 1–7) and were studied at a median postnatal age of 5 (range 4–10) days. One infant had a right-sided defect. One infant had a fetal endoscopic tracheal occlusion sited (FETO), which was removed in utero at 34 weeks of gestation. Two infants were diagnosed with CDH postnatally. The antenatally observed/expected lung-to-head ratio (LHR) was available for six infants, with a median LHR of 42% (range 24–55%). Seven infants underwent a primary surgical repair, two had a patch repair. The measured median (range) tidal volume at baseline was 5.25 (2.92–7.87) ml/kg (Table 1).

**Table 1:** Infant characteristics

Gestational age at birth (weeks)	38 + 4 (36 + 4–40 + 6)
Sex (male)	8 (89%)
Birth weight	3202 (2855–3800) g
Site of CDH (left)	8 (89%)
Worst LHR – available for 6/9	42% (24–55%)
Surgical day of repair	4 (range 1–7)
Type of repair: Primary Patch	7 (78%) 2 (22%)

Results presented as median (range) or *n* (%)

**Fig. 1:**
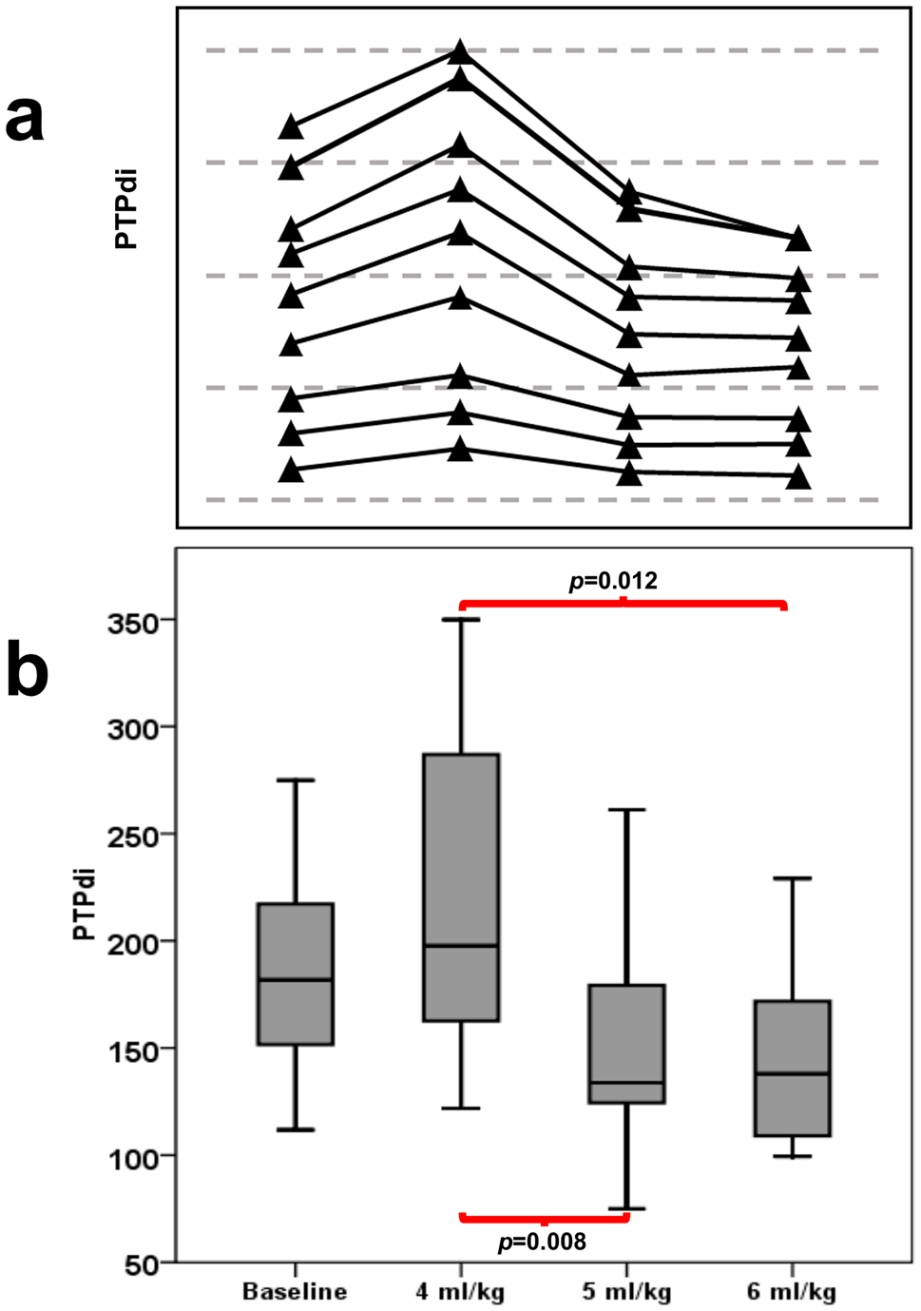
Comparison of the PTPdi results at the different volume targeted levels. Staggered results for individual infants **a** and boxplots **b** are presented. The horizontal lines in the boxes represent the median and lower and upper quartile values

The PTPdi at 4 ml/kg VTV was higher than at baseline in all infants studied (Fig. 1a and b) and there was a reduction in the WOB at both 5 and 6 ml/kg compared to 4 ml/kg (Table 2). Post hoc analysis revealed that the PTPdi was higher at 4 ml/kg than at both 5, *p* = 0.008, and 6 ml/kg, *p* = 0.012. There was no significant difference between the PTPdi at 5 and 6 ml/kg, *p* = 0.263.

**Table 2:** Results

	Baseline	4 ml/kg	5 ml/kg	6 ml/kg
PTPdi cmH_2_Os/min	181.7 (111.8–274.9)	197.67 (161.6–349.9)	133.73 (118.6–261.2)	137.91 (99.5–229.1)
PIP (cmH_2_O)	19.47 (10.9–25.47)	10.55 (8.8–23.92)	16.58 (10.1–29.06)	20.43 (8.9–35.09)
RR (breaths/min)	45 (29–69)	54 (31–68)	43 (30–54)	40 (31–58)

Results presented as median (range)

## Discussion

We have demonstrated that the work of breathing was significantly higher when ventilating CDH infants post-operatively with a targeted tidal volume of 4 ml/kg compared to 5 or 6 ml/kg. There was no significant difference in the work of breathing between 5 and 6 ml/kg. We should note that the PTPdi is an index of the work of breathing and thus might not be the only parameter to decide on the optimal tidal volume during mechanical ventilation and is not a surrogate guide to lung protective mechanical ventilation. It should be noted that the goal of respiratory support is not necessarily to alleviate all the work of breathing. In fact, preservation of a substantial contribution of the infant to the overall tidal volume is desirable, as long as the total work of breathing is not excessive and acceptable gas exchange is achieved. Negative intrathoracic pressure generated during spontaneous breathing facilitates venous return and mitigates the adverse hemodynamic consequences of positive pressure ventilation. This is the main reason we usually avoid muscle relaxation during neonatal ventilation.

Previous studies have reported mean values of the PTPdi of 141 cm H_2_Os/min in spontaneously breathing infants on synchronised intermittent mandatory ventilation [18]. We report values in the range of 133–197 H_2_Os/min. The overall higher values in our study might be explained by the disease itself as the diaphragmatic work of breathing would be impaired by the diaphragmatic defect and the ensuing decreased muscle mass. The PTPdi measurements should be taken in the context of other clinical signs of respiratory distress such as visible intercostal or subcostal recessions. The median respiratory rate in all volume target levels in our study was between 40 and 55 breaths per minute suggesting that the infants were not in marked respiratory distress.

The use of volume-targeted ventilation (VTV) is a lung protective strategy which aims to avoid too high or too low delivered volumes to an infant’s lungs [11]. It is clear that infants born with CDH are particularly at risk of ventilator-induced lung injury as a result of their hypoplastic lungs and disparity between the ipsilateral and contralateral lungs [19]. Volume-targeted ventilation (VTV) is usually set on clinical assessment based on an infant’s size. As CDH infants, however, have abnormal lung development with varying degrees of pulmonary hypoplasia, the assumption may be that lower targeted volumes would be appropriate compared to unaffected infants of the [20]. Of note, te Pas et al. studied 12 infants with CDH receiving respiratory support at birth and reported that the mean tidal volume was significantly different for spontaneous breaths (3.8 ml/kg), spontaneous breaths coinciding with manual inflation (4.7 ml/kg), and manual inflations alone (2.6 ml/kg) (20).The results of our study are in keeping with a retrospective study of infants with CDH who were managed with conventional ventilation post-operatively in whom mean tidal volumes of 4.53 (± 0.79) ml/kg were required to maintain adequate PaCO_2_ values [21].

Within our study, two infants were diagnosed postnatally, which often confers improved outcomes [22], and five of six with a known LHR met the classification of having mild-moderate CDH [23]. It is, therefore, not appropriate to extrapolate as to whether infants with more severe disease with a greater degree of pulmonary hypoplasia would benefit from lower tidal volumes than our findings present.

In conclusion, assisted ventilation at a targeted tidal volume of 4 ml/kg was associated with an increased work of breathing compared to 5 and 6 ml/kg, suggesting that a tidal volume of 4 ml/kg should be avoided in post-operatively spontaneously breathing patients with mild-to-moderate CDH. Although the work of breathing in our study was lower for targeted volumes of 5 and 6 ml/kg, the optimal volume target should be individualised for each subject as maybe there is no blanket correct tidal volume for all infants. It is unclear whether targeting a Vt of 5 ml/kg will be best for all patients in terms of a lung protective ventilation strategy, but we suggest that a tidal volume of 5 ml/kg would be a good starting point for most infants, subject to subsequent revision as the individual clinical response dictates.

## Data Availability

Data provided on reasonable request.

## Abbreviations

CDH: Congenital diaphragmatic hernia
FETO: Fetal endoscopic tracheal occlusion
LHR: Lung-to-head ratio
PTPdi: Pressure-time product of the diaphragm
VTV: Volume targeted ventilation
WOB: Work of breathing

## Declarations

All procedures performed in studies involving human participants were in accordance with the ethical standards of the institutional and/or national research committee and with the 1964 Helsinki declaration and its later amendments or comparable ethical standards.

### Disclaimer

The views expressed are those of the authors and not necessarily those of the NHS, the NIHR, or the Department of Health.
